# Supplementary material for: Comprehensive Analysis of the Yield and Leaf Quality of Fresh Tea (Camellia sinensis cv. Jin Xuan) under Different Nitrogen Fertilization Levels
Source: Foods. 2024 Jul 1;13(13):2091. doi: 10.3390/foods13132091 (PMC11241149; doi:10.3390/foods13132091)
Supplement: Supplementary file 1 [file foods-13-02091-s001.zip › foods-3030647-supplementary.pdf]

Table S1. Sequences of primers used for the qRT-PCR analysis.

Gene name	Forward primer (5'→3')	Forward primer (3'→5')
PAL	CTCCAATTCCTTGCCAATCCTG	CAACTGCCTCGGCTGTCTTTCT
LAR	TGCAGCCTCTACCCTGATGA	TCTGGGCAGTGTTTCCATCC
DFR	AGTTGTGTCGTTCTCATC	GTATCAATGGCTCCTCTG
CsTCS1	AGCAAAGCTACCGAAGACCA	TCCACACAAGAGCAAAATGC
CsSAM1	CTTACGCCATTGGTGTTCCT	GGCAGCAGTCTTCAAGAACC
CsTSI	GTTGATGTTTCTGGGCAGCA	CTCACCCACACCAGTCAGAT
TCS1	CTGTCGTCTGAGGTTATTGG	GTGCCTGAGTAAGCCAATGA
CsHEMA1	TGCAGCTGACAGGTATACAAA	ATGGCAAGCTTTTCACGCATT
CsHEMA2	GGAAGTTGGGGGTCTTAGGC	CGTTATAAACCCGTGTGGCG
GOGAT	AAGGTGCAAAGCCTGGTGAA	TCAGTTGGGCAAGATCCTCG

Table S2. PCR Reaction Program.

	Temperature	Time	Other
Program 1	95℃	5min	
Program 2<br>40 cycles	96℃	10s	
	55℃	10s	
	72℃	30s	Signal collection
Program 3	95℃	30s	Collection of dissolution curve
	65℃	1min	
	95℃	Continuous	
Program 4	40℃	1min	Slow cooling

Table S3. Parameters of the calibration curves for the components and their limits of detection (LOD) and limits of quantification (LOQ).

Name	Equation	R^2	LOD mg/L	LOQ mg/L
Tea polyphenols	Y = 0.0112x+0.0265	0.9905	3.8389	12.7963
Free amino acid	Y = 1.98x-0.2834	0.9993	0.0141	0.0469
Caffeine	Y = 5.80e+007 X + 9.45e+004	0.9984	0.0001	0.0002
Theanine	Y = 9.17e+006 X - 1.49e+004	0.9928	0.0014	0.0048
GC	Y = 2.03e+006 X + 6.88e+003	0.9872	0.0392	0.1308
EGC	Y = 1.82e+006 X + 1.61e+005	0.9874	0.0761	0.2536
EC	Y = 7.43e+006 X + 1.13e+006	0.9757	0.0856	0.2852
EGCG	Y = 1.47e+007 X + 5.85e+005	0.9942	0.0686	0.2285
GCG	Y = 1.63e+007 X + 6.20e+005	0.9981	0.0653	0.2176
ECG	Y = 1.62e+007 X + 1.88e+006	0.9775	0.0557	0.1855

Table S4. Effects of nitrogen levels on main biochemical components and yield of fresh tea leaves in summer.

Name	Summer			
	N0	N150	N300	N450
TP	269.48±0.49c	321.44±1.58a	265.45±0.80d	279.70±0.61b
AA	34.52±0.13a	30.22±0.05c	32.47±0.05b	32.41±0.05b
TP/AA	7.81±0.02d	10.64±0.04a	8.18±0.02c	8.63±0.02b
TC	129.92±0.39b	142.53±1.55a	130.15±0.51b	128.88±0.55b
Chla	1.02±0.01b	1.00±0.02b	1.02±0.01b	1.15±0.01a
Chlb	0.36±0.01b	0.36±0.03b	0.34±0.02b	0.51±0.02a
Chla+b	1.37±0.02b	1.36±0.05b	1.36±0.02b	1.66±0.02a
Chla/b	2.86±0.06a	2.77±0.17a	3.02±0.26a	2.26±0.06b
Caffeine	30.3±0.30b	31.2±0.00a	28.2±0.00c	26.80±0.17d
Theanine	3.30±0.00b	2.60±0.10d	2.83±0.06c	3.73±0.06a
EGCG	69.27±0.23b	72.55±0.64a	67.31±0.12c	67.25±0.18c
ECG	16.01±0.05b	18.19±0.18a	14.83±0.06c	13.81±0.13d
EGC	34.61±0.16d	39.99±0.51a	37.23±0.4c	38.43±0.12b
EC	4.12±0.07b	4.61±0.15a	4.35±0.05b	3.44±0.11c

GCG	4.44±0.00c	5.36±0.07a	4.83±0.06b	4.16±0.07d
GC	1.47±0.02c	1.84±0.04a	1.61±0.02b	1.79±0.02a
Yields	41.07±4.40b	45.13±5.41ab	55.43±7.05a	47.40±0.98ab

The lowercase letters in the Tables are the marks of significant differences, and different letters indicate significant differences.

Table S5. Effects of nitrogen levels on main biochemical components and yield of fresh tea leaves in autumn.

Name	Autumn			
	N0	N150	N300	N450
TP	180.67±6.03c	207.33±6.11a	219.33±1.53a	194.00±3.00b
AA	31.29±1.86b	35.25±0.57a	37.80±1.48a	35.89±1.76a
TP/AA	5.80±0.55a	5.88±0.23a	5.81±0.19a	5.42±0.33a
TC	109.97±0.46a	109.40±0.07a	104.71±0.51c	106.84±0.59b
Chla	1.14±0.01a	1.11±0.01b	1.03±0.01c	1.04±0.01c
Chlb	0.51±0.02a	0.62±0.02b	0.46±0.01c	0.39±0.01d
Chla+b	1.65±0.03b	1.73±0.02a	1.50±0.02c	1.43±0.02c
Chla/b	2.24±0.07b	1.77±0.03c	2.24±0.05b	2.63±0.08a
Caffeine	27.6±0.00d	29.30±0.17b	30.6±0.00a	28.8±0.00c
Theanine	5.77±0.15c	7.10±0.00b	7.87±0.06a	6.97±0.06b
EGCG	57.37±0.02a	56.45±0.08b	56.24±0.21b	57.12±0.17a
ECG	7.85±0.09b	8.04±0.00a	7.71±0.08b	8.12±0.07a
EGC	35.95±0.30b	37.57±0.02a	34.56±0.04c	35.03±0.28c
EC	2.87±0.05a	2.76±0.00a	1.96±0.07b	1.99±0.05b
GCG	4.16±0.28a	2.68±0.08b	2.59±0.36b	3.00±0.00b
GC	1.77±0.05ab	1.89±0.06a	1.65±0.02bc	1.59±0.05c
Yields	50.9±10.00c	81.10±0.90b	103.70±3.50a	81.90±1.30b

The lowercase letters in the Tables are the marks of significant differences, and different letters indicate significant differences.

Table S6. VIP value of non-volatile compounds

Name	VIP value (summer)	VIP value (autumn)
TP	1.91	1.91
EGC	1.22	1.37
Caffeine	1.15	0.58
TC	1.12	1.26
AA	1.10	1.41
Theanine	0.96	0.52
EGCG	0.89	0.59
ECG	0.86	0.97
EC	0.85	0.89
GCG	0.77	0.87
Chlb	0.36	0.38
GC	0.32	0.50
Chla	0.30	0.26
